# CH_3_NH_3_PbI_3_ grain growth and interfacial properties in meso-structured perovskite solar cells fabricated by two-step deposition

**DOI:** 10.1080/14686996.2017.1298974

**Published:** 2017-04-10

**Authors:** Zhibo Yao, Wenli Wang, Heping Shen, Ye Zhang, Qiang Luo, Xuewen Yin, Xuezeng Dai, Jianbao Li, Hong Lin

**Affiliations:** ^a^ State Key Laboratory of New Ceramics & Fine Processing, School of Materials Science and Engineering, Tsinghua University, Beijing, PR China; ^b^ National Engineering Laboratory for Modern Silk, College of Textile and Clothing Engineering, Soochow University, Suzhou, PR China; ^c^ State Key Laboratory of Marine Resource Utilization in South China Sea, Materials and Chemical Engineering Institute, Hainan University, Haikou, PR China

**Keywords:** Perovskite solar cell, two-step deposition, CH_3_NH_3_PbI_3_ grain growth, interfacial property, concentration of PbI_2_, 50 Energy Materials, 209 Solar cell / Photovoltaics, 302 Crystallization / Heat treatment / Crystal growth

## Abstract

Although the two-step deposition (TSD) method is widely adopted for the high performance perovskite solar cells (PSCs), the CH_3_NH_3_PbI_3_ perovskite crystal growth mechanism during the TSD process and the photo-generated charge recombination dynamics in the mesoporous-TiO_2_ (mp-TiO_2_)/CH_3_NH_3_PbI_3_/hole transporting material (HTM) system remains unexploited. Herein, we modified the concentration of PbI_2_ (*C*_(PbI2)_) solution to control the perovskite crystal properties, and observed an abnormal CH_3_NH_3_PbI_3_ grain growth phenomenon atop mesoporous TiO_2_ film. To illustrate this abnormal grain growth mechanism, we propose that a grain ripening process is taking place during the transformation from PbI_2_ to CH_3_NH_3_PbI_3_, and discuss the PbI_2_ nuclei morphology, perovskite grain growing stage, as well as Pb:I atomic ratio difference among CH_3_NH_3_PbI_3_ grains with different morphology. These *C*_(PbI2)_-dependent perovskite morphologies resulted in varied charge carrier transfer properties throughout the mp-TiO_2_/CH_3_NH_3_PbI_3_/HTM hybrid, as illustrated by photoluminescence measurement. Furthermore, the effect of CH_3_NH_3_PbI_3_ morphology on light absorption and interfacial properties is investigated and correlated with the photovoltaic performance of PSCs.

## Graphical abstract

**Figure:**
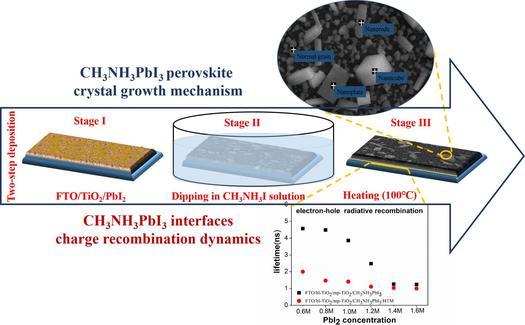

## 1. Introduction

The scientific community has witnessed an unprecedented and unexpected technological advance in the field of photovoltaics brought about by the advent of an organometal halide perovskite material, CH_3_NH_3_PbX_3_ (X = Cl, Br, or I) [1–7]. Significant progress in perovskite solar cells (PSCs) has been achieved in the past three years, and a certified power conversion efficiency (PCE) up to 22% has been recently reported [8]. The stunning success to apply organometal halide perovskite in the context of photovoltaics would not have been realized without the optimization of highly crystallized perovskite layer. A desirable perovskite thin film would exhibit a complete surface coverage with no defects, a small surface roughness and a well-defined grain structure [9–11]. Meanwhile, devices with mesoporous structure are widely investigated because of the minimized carrier drift and diffusion length [12,13], which benefits from the direct electron injection from perovskite to electron-transporting materials (ETM). The electron transport layers in meso-structured devices usually employ wide bandgap semiconductors, such as TiO_2_ [14,15], and ZnO [16]; the crystallization of the perovskite material also benefits greatly from the enhanced nucleation at the meso-structure interfacial sites. In addition, insulators such as ZrO_2_[17] or Al_2_O_3_[18] are also applied as scaffold in PSCs, where the light induced electrons are generated and transported through the perovskite material itself and then injected to charge the selective layer directly.

Based on these fundamental architectures, versatile film formation technologies such as vacuum evaporation [19], sequential deposition [14], and vapor-assisted deposition[20] were developed for efficient PSC fabrication. In these previous studies, a significant development in perovskite fabricating process is the two-step deposition (TSD) method, originally applied and developed by Liang and Burschka et al.[14,21] The pre-formed PbI_2_ particles react with CH_3_NH_3_I in isopropanol solution via *in situ* insertion reaction, leading to full film coverage by CH_3_NH_3_PbI_3_ formation. This approach was proved to be effective in ensuring better repeatability by restraining the PbI_2_ crystals within the diameter of the TiO_2_ mesopores and facilitating the reaction with CH_3_NH_3_I. The TSD technique has been adopted by many groups for high-performance PSC fabrication, and various optimizations have been carried out through solution chemistry/processing modification, such as additive-effects on PbI_2_ solution [22], retarding PbI_2_ crystallization using dimethyl sulfoxide solvent [23] and PbI_2_ morphology optimization [24–26]. Park et al. [27] applied thermodynamics to correlate the crystal size with the concentration of CH_3_NH_3_I and reaction temperature. However, the exact perovskite crystal growth mechanism during the TSD and the detailed free charge recombination dynamics on the TiO_2_/perovskite interface or within the bulk of semiconductor remain unexploited. Moreover, controlling perovskite morphology is critical for developing high efficient devices. The coverage of perovskite film over mesoporous TiO_2_ film, perovskite crystallinity and surface roughness can dramatically affect the photoelectrochemical properties, such as light harvesting, charge carrier transport and charge dissolution across the interfaces [28–30].

In this work, we report an abnormal CH_3_NH_3_PbI_3_ grain growth atop mesoporous TiO_2_ film during concentration variation of PbI_2_ (*C*_(PbI2)_) using TSD technique. Corresponding to different perovskite morphologies, the photo-generated charge recombination mechanism of the mesoporous-TiO_2_ (mp-TiO_2_)/CH_3_NH_3_PbI_3_/hole transporting materials (HTM) system is characterized by photoluminescence [31] measurement. Typical *C*_(PbI2)_-dependent perovskite morphology shows a significant effect on the light-harvesting, interface properties and photovoltaic performance of the as-made PSCs.

## 2. Experimental section

### 2.1. Materials

All the solvents and reagents were used as received, including PbI_2_ (99%, Sigma-Aldrich, Darmstadt, Germany), hydroiodic acid (HI, 57 wt% in water, Sigma-Aldrich, Taiwan, China), methylamine (CH_3_NH_2_, 33 wt% in ethanol, Sigma-Aldrich), spiro-OMeTAD (Luminescence Technology Corp., Heysham, UK)), lithium bis(trifluoromethylsulfon)imide (LiTFSI, Alfa Aesar), 4-tert-butylpyridine (TBP, 96%, Sigma-Aldrich). Conductive fluorine-doped tin oxide glasses (FTO, sheet resistance of 15 Ω/ sq. Nippon Sheet Glass Co., Osaka, Japan) were applied as substrates.

### 2.2. Device fabrication

CH_3_NH_3_I was produced according to a published method [32]. Briefly, CH_3_NH_3_I was synthesized by dropping HI into CH_3_NH_2_ solution at 0 °C under continuous stirring. By evaporating the ethanol at 100 °C, the precipitate was collected. Then the CH_3_NH_3_I powders were recrystallized from ethanol and washed with diethyl ether three times and finally dried in a vacuum oven before use.

FTO glasses were cleaned sequentially by ultra-sonication in detergent, deionized water, ethanol, acetone and isopropanol and dried with nitrogen and then cleaned by UV/ozone before use. A compact TiO_2_ blocking layer (bl-TiO_2_) was firstly deposited onto the surface of FTO substrate by spin-coating titanium isopropoxide in ethanol with HCl additives as hydrolysis inhibitor. The film was then sintered at 500 °C for 30 min [33]. An mp-TiO_2_ layer was then fabricated by spinning TiO_2_ paste (Dyesol 18 NR-T, 18 nm average nanoparticle diameter) diluted in anhydrous ethanol (2:7 by weight) at 5000 rpm for 60 s. The layers were then sintered in the oven at 500 °C for 30 min. Prior to use, the films were again dried at 110 °C for 30 min to remove water.

A PbI_2_ solution in dimethylformamide (DMF; 0.6 M/ 0.8 M/ 1.0 M/ 1.2 M/ 1.4 M/ 1.6 M) was then deposited onto the mp-TiO_2_ film by spin-coating at 6000 rpm for 60 s. The PbI_2_ films were dried at 100 °C on a hotplate for 30 min. After gradually cooling to room temperature, the films were dipped in a 10 mg ml^–1^ CH_3_NH_3_I in iso-propanol solution for 20 min to obtain CH_3_NH_3_PbI_3_, rinsed with 2-propanol for 15 s and then dried at 100 °C in a vacuum oven for 30 min.

The HTM solution was prepared by dissolving 72.3 mg spiro-OMeTAD, 28.8 μl TBP and 17.5 μl of a solution of 520 mg ml^–1^ LiTFSI in acetonitrile into 1 ml anhydrous chlorobenzene. The FTO/bl-TiO_2_/mp-TiO_2_/CH_3_NH_3_PbI_3_ film was covered with HTM solution by spin-coating at 4000 rpm for 60 s. Devices were kept in a dry box (air humidity < 10%) for 10 h prior to thermal evaporation of 80 nm Au electrodes (under ~10^−6^ Torr vacuum, at a rate of ~ 6 nm min^–1^). All spin-coating operations were carried out in the air condition with a humidity of < 30%.

### 2.3. Film and device characterization

The surfaces and cross-sections of the samples were observed with a field emission scanning electron microscope (FESEM, LEO 1530 Gemini, Zeiss, Oberkochen, Germany). The crystal structure of the samples was characterized with a X-ray diffractometer (XRD, Bruker D8, Billerica, MA, USA) using Cu Kα radiation (1.54 Å). UV-vis reflectance and transmittance spectra were obtained by an UV/vis/NIR spectrophotometer (Lambda 950, Perkin-Elmer, Waltham, MA, USA) equipped with an integrating sphere module. To simulate the cells’ working condition, the monochromatic light was illuminated through the FTO side of samples. The incident photon to current efficiency (IPCE) was measured with a quantum efficiency characterization system (QEX10, PV measurements, Boulder, CO, USA). Prior to starting the measurement, a standard silicon solar cell as the reference was used to calibrate the spectra from 300 to 1000 nm. Steady-state and time-resolved PL spectra were both acquired with an optical spectrometer (FLS920, Edinburgh Instruments, Livingston, UK). The steady-state PL spectra were collected by illuminating the sample with a monochromatic light (λ_exc_=460 nm, xenon lamp). The time-resolved PL spectra were acquired with samples excited by a pulsed laser beam (405 nm, 10 MHz, pulse duration of < 100 ps) under a time-correlated single photon counting (TCSPC) mode. PL lifetime was deduced by fitting the collected transient curves with a bi-exponential function as shown below:

(1) $$\text{I}\left(\text{t}\right)=A_{1}\text{exp}\left(-\text{t}/\tau_{1}\right)+A_{2}\text{exp}\left(-\text{t}/\tau_{2}\right)$$

The current density-voltage (*J*−*V)* characteristics of the cells were collected under illumination of a solar simulator (AM 1.5, 1000 W/m^2^, Oriel, Irvine, CA, USA), calibrated with a standard silicon solar cell (Si PDS1337–1010BQ, Bunkoukeiki Co., Ltd., Tokyo, Japan) with a Keithley 2400 digital source meter (Keithley, Solon, OH, USA). The scanning voltage step during the measurement is 10 mV, and the dwell time at each voltage is 100 ms. Forward and backward scans were carried out between 1.2 V and –0.1 V.

## 3. Results and discussion

**Figure 1.**
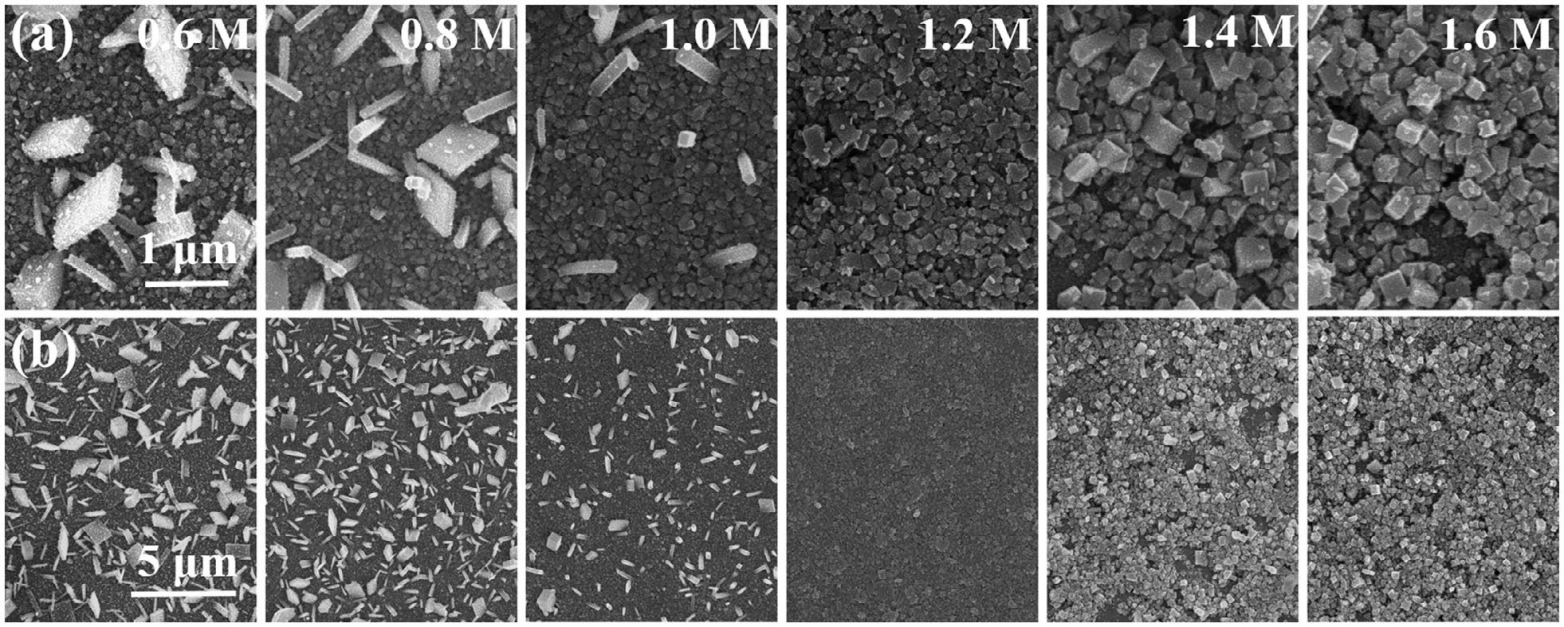
(a) FESEM top views of FTO/bl-TiO_2_/mp-TiO_2_ /CH_3_NH_3_PbI_3_ films fabricated with *C*_(PbI2)_ of 0.6, 0.8, 1.0, 1.2, 1.4 and 1.6 M, (b) low magnification images of (a).

The FTO/bl-TiO_2_/mp-TiO_2_/CH_3_NH_3_PbI_3_ films with different morphologies were successfully fabricated by the typical TSD method with varied *C*_(PbI2)_, ranging from 0.6 M to 1.6 M. Top-view SEM images of the samples are shown in Figure 1. It can be observed that the mp-TiO_2_ layer has been fully covered by the CH_3_NH_3_PbI_3_ layer (Figure 1(a), 1(b)) at all conditions, when a relatively high spinning speed (6000 rpm) for PbI_2_ solution was used. We also found that higher spinning speed is better for the homogeneous spreading of PbI_2_ on top of the mesoporous structure (Figure S1). The thickness of the PbI_2_ layer was gradually increased from 78 to 471 nm with the increased *C*_(PbI2)_ of 0.6 M to 1.6 M, as shown in Figure S2. The PbI_2_ layer was of porous morphology at high *C*_(PbI2)_ which was caused by the evaporation of excess DMF when annealing. When immersing PbI_2_ film into CH_3_NH_3_I solution, densely packed layers consisted of crystalline perovskite particles were formed, although the perovskite coverage seemed to be less complete when increasing the *C*_(PbI2)_ up to 1.4 M and 1.6 M, which may be caused by the strong driving force of nucleation at such a high concentration and the non-uniform evaporation of DMF in the presence of thick capping layer [34].

**Figure 2.**
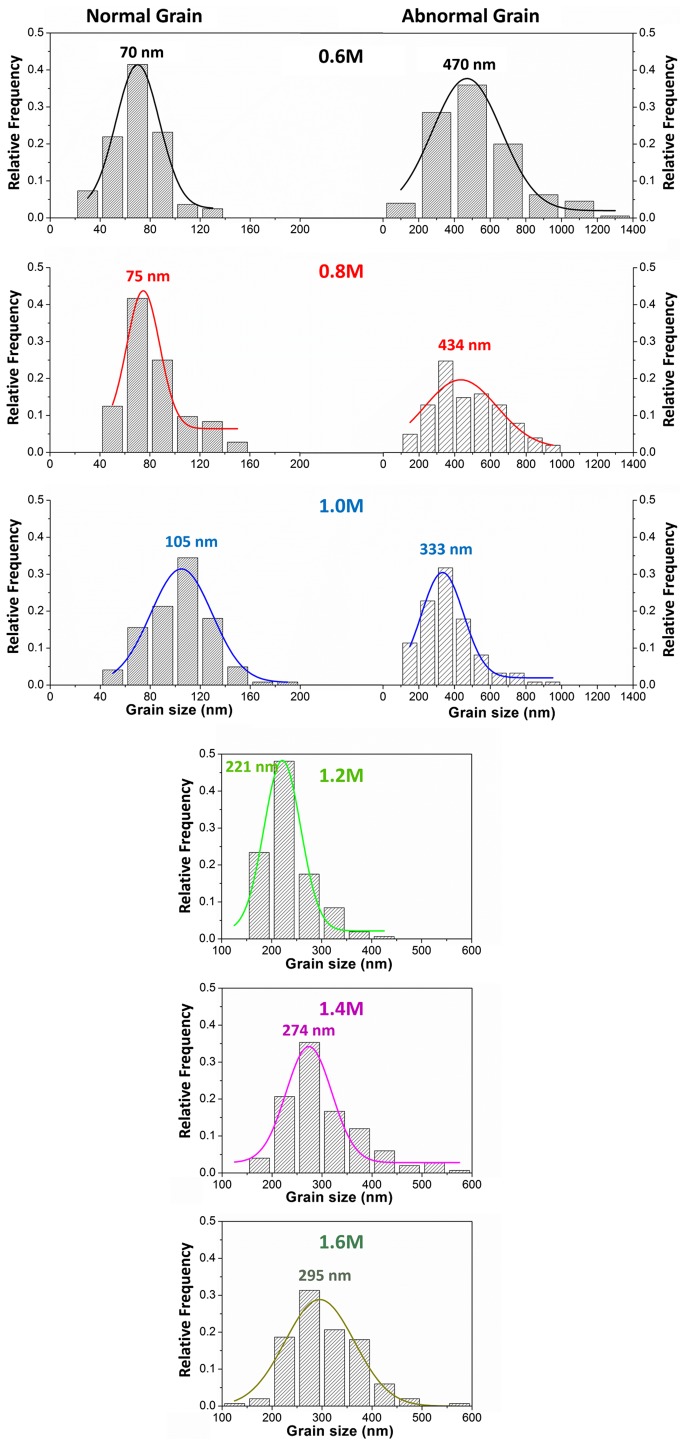
Statistical distributions and their Gaussian fitting (solid lines) of the CH_3_NH_3_PbI_3_ grain sizes determined from the FESEM images. For the samples of 0.6, 0.8 and 1.0 M, the grains with normal sizes and the abnormal grains were collected separately. No abnormal grain occurred in samples of 1.2, 1.4 and 1.6 M, so they showed only one peak in their size distributions.

More importantly, we observed an abnormal grain growth of perovskite crystals, especially at low *C*_(PbI2)_, with morphologies of nanocubes, nanoplates or nanorods, hundreds nanometers in size, randomly distributed on the mp-TiO_2_. The average grain size of the abnormally grown big crystals versus *C*_(PbI2)_ was summarized in Figure 2, which revealed that the grain size distributions gradually centralized when *C*_(PbI2)_ was increased from 0.6 M to 1.0 M. The abnormal crystals disappeared completely when *C*_(PbI2)_ increased beyond 1.2 M, while the average crystal size increased from 221 to 274 and to 295 nm respectively at *C*_(PbI2)_ of 1.2, 1.4 and 1.6 M, as determined by Gaussian fitting of the grain size distributions. We consider the abnormal perovskite grain growth at low *C*_(PbI2)_ to be the result of the relatively high surface energy of small crystalline grains, which leads to crystalline grains growing or ‘ripening’ at the expense of others, so as to reduce grain boundary energy during the transformation of PbI_2_ to CH_3_NH_3_PbI_3_.

As illustrated in Figure S3, we separate the abnormal grain grown process of CH_3_NH_3_PbI_3_ into three stages. During stage I, the spin-coated PbI_2_ layer at *C*_(PbI2)_ of 0.8 M showed a homogeneous crystalline distribution with the absence of abnormally grown grains (Figure S3(a)). In stage II, the layered PbI_2_ structure, consisting of three spatially repeating planes (I–Pb–I) [35], facilitates the insertion of the CH_3_NH_3_I to form the hybrid perovskite accompanied by a volume expansion from the originally edge-sharing octahedral structure to the corner-sharing octahedral structure in perovskite films [36]. In the meantime, the abnormal grain growth occurred during this stage as we observed the existence of CH_3_NH_3_PbI_3_ crystals with a diameter of hundreds of nanometers (Figure S3(b)) when the samples were taken out of CH_3_NH_3_I solution without further annealing. We think that the abnormal grain growth may be driven by the reduction of boundary length, which helps to minimize the surface free energy. The abnormal grain growth is a grain ripening process, and this crystalline rearrangement often appears during polycrystalline film growth [37]. Different PbI_2_ concentrations strongly affect the density of PbI_2_ nuclei at Stage I and further on the kinetic process of perovskite nucleation in the initial transforming stages. Meanwhile, the broad distribution of grain size especially at low *C*_(PbI2)_ may also contribute to the local concentration differences, which thus resulted in the diffusion-controlled mass transport and gave rise to an abnormal grain growth [37]. As an evidence of the concentration difference, we characterized the element composition of the normal CH_3_NH_3_PbI_3_ crystals and the abnormal ones with different morphologies using energy dispersive spectroscopy (EDS) measurement in SEM as shown in Figure S4. The Pb:I atomic ratios were 1:2.80 ± 0.20, 1:2.82 ± 0.14 and 1:2.77 ± 0.12 for abnormal big-size CH_3_NH_3_PbI_3_ crystals with shapes like nanocubes, nanoplates and nanorods respectively, which showed that the I vacancy or I substituted by Pb occurred in the big-size crystals comparing to the ideal 1:3 ratio of Pb:I atom for CH_3_NH_3_PbI_3_ crystal. As for the normal CH_3_NH_3_PbI_3_ crystals with size of 50–100 nm, a Pb:I atomic ratio of 1:3.39 ± 0.31 was found. The significant difference of Pb:I atomic ratio in different CH_3_NH_3_PbI_3_ crystal morphologies proved the mass transport effect on the abnormal grain growth. As for *C*_(PbI2)_ over 1.2 M, the PbI_2_ reactant and the density of nuclei of perovskite grains are sufficient enough to form a homogenous perovskite capping layer with a narrow distribution of grain size. Stage III is the annealing process, during which the residual solvent evaporated and the CH_3_NH_3_PbI_3_ crystals grew further. As shown in Figure S3(c) and S3(d), the integral CH_3_NH_3_PbI_3_ morphology did not significantly change, in spite of a slight grain growth during annealing time of 30 min at 100 °C.

The optical properties of the perovskite films with typical *C*_(PbI2)_-dependent morphologies and as-made devices were characterized as shown in Figure S5, including the transmittance (T), reflectance (R) and absorbance (A) of FTO/bl-TiO_2_/mp-TiO_2_/CH_3_NH_3_PbI_3_ films. Absorbance was calculated as A=1 – T – R.

PbI_2_ films with typical yellow color (Figure S5, inset) turned darker and more opaque with PbI_2_ concentration increasing from 0.6 M to 1.6 M. Meanwhile, the as-prepared CH_3_NH_3_PbI_3_ films exhibit strong light absorption from 350 to 780 nm, whereas the light absorption is relatively weak from 550 to 780 nm, indicating inadequate light harvesting (Figure S5(c)). Notably, the absorbance of the FTO/bl-TiO_2_/mp-TiO_2_ /CH_3_NH_3_PbI_3_ film increased significantly from 0.6 to 1.2 M, and reached a plateau when *C*_(PbI2)_ was 1.2 M, where the abnormal big-size crystals disappeared completely (Figure 1). However, when the concentration was further increased to 1.6 M, a slight increase of absorbance occurred, which could be attributed to the thickening of the perovskite capping layer, as illustrated in Figure 1.

**Figure 3.**
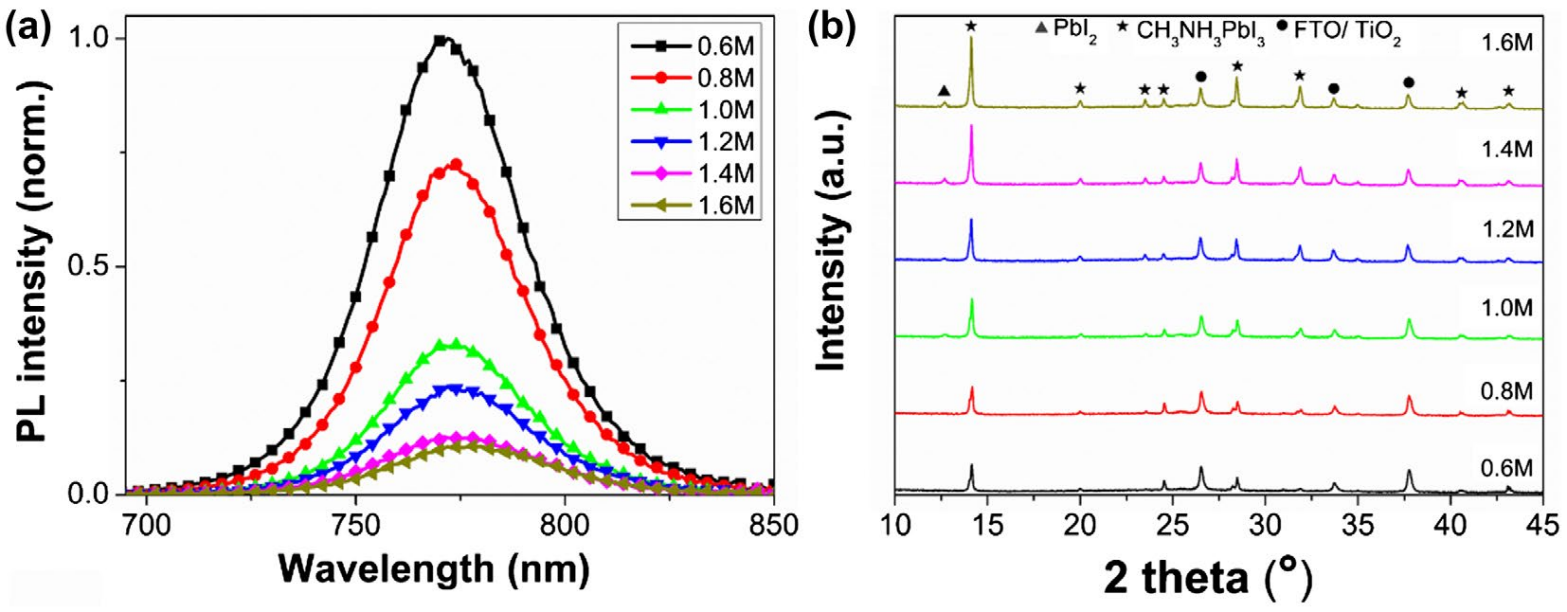
(a) Steady-state PL spectra and (b) XRD patterns of FTO/bl-TiO_2_/mp-TiO_2_ /CH_3_NH_3_PbI_3_ films as a function of *C*_(PbI2)_.

The charge separation and transport in perovskite layers happened when the free electrons were excited to the conduction band of perovskite by the absorption of light, which was then investigated by the steady-state and time-resolved PL measurement associated with *C*_(PbI2)_-dependent morphologies. An emission peak around 770 nm was detected for all the samples. We found that the PL intensity decreased when increasing *C*_(PbI2)_ from 0.6 M to 1.6 M (Figure 3(a)), despite more perovskite loading at higher *C*_(PbI2)_. This is in stark contrast to the work by Bi et al. [25] where the weak PL emission indicates a lower amount of perovskite. The less light absorption for lower amount of perovskite observed by Bi et al. indicates that a portion of the exciting light is scattered elsewhere. This should result in sample-dependent light absorption, whereas we believe that PL intensity reflects the real recombination mechanisms in perovskite films only when the exciting light is equally absorbed by the samples. For samples of 1.0 to 1.6 M, we could find the complete absorption of the exciting light of 460 nm (as shown in Figure S5(c)), so that the lower PL emission at higher *C*_(PbI2)_ indicated a larger quenching efficiency of photo-generated charges corresponding to a more effective electron injection across the interface between TiO_2_ and perovskite. The sample of 0.6 M, with inadequate absorption of 460 nm light (Figure S5(c)), however showed very strong PL emission. This further proved the lower quenching efficiency at lower *C*_(PbI2)_. It is worth noting that the PL decline may also due to the intrinsic light emission behavior of the samples with different *C*_(PbI2)._ In bare semiconductor film, the PL intensity will be affected by the non-radiative recombination at energetically favored sites such as discrete defect and impurity sites at interfaces and within the bulk of the material [38]. However, the effect of crystal morphology and defect on the perovskite PL intensity is still unclear. The PL intensity varied a lot even for the different crystals in one perovskite film as shown in the PL mapping measurement given by de Quilettes et al. [39]. The discussion on the time-resolved PL spectra in the following text also illustrates the quenching effect of the interface between ETM and perovskite.

The XRD patterns of the CH_3_NH_3_PbI_3_ using different *C*_(PbI2)_ were also characterized, and are shown in Figure 3(b). The strong Bragg peaks at 14.08, 28.41, 31.85, and 43.19° can be assigned to (110), (220), (310), and (330) planes of the CH_3_NH_3_PbI_3_ phase, which is in good agreement with literature data corresponding to a tetragonal I4 cm crystal structure of halide perovskite [40]. A small peak at 12.65° was identified for the samples above 1.0 M, which belongs to the (001) lattice plane of PbI_2_, indicating incomplete phase conversion [14], which could be attributed to the PbI_2_ layer becoming more compact when *C*_(PbI2)_ is higher than 1.0 M [25]. Such PbI_2_ impurity has been widely observed even in highly efficient devices reported by other groups, which was claimed to have a positive effect on the cell performance by passivating the perovskite layers [41].

**Figure 4.**
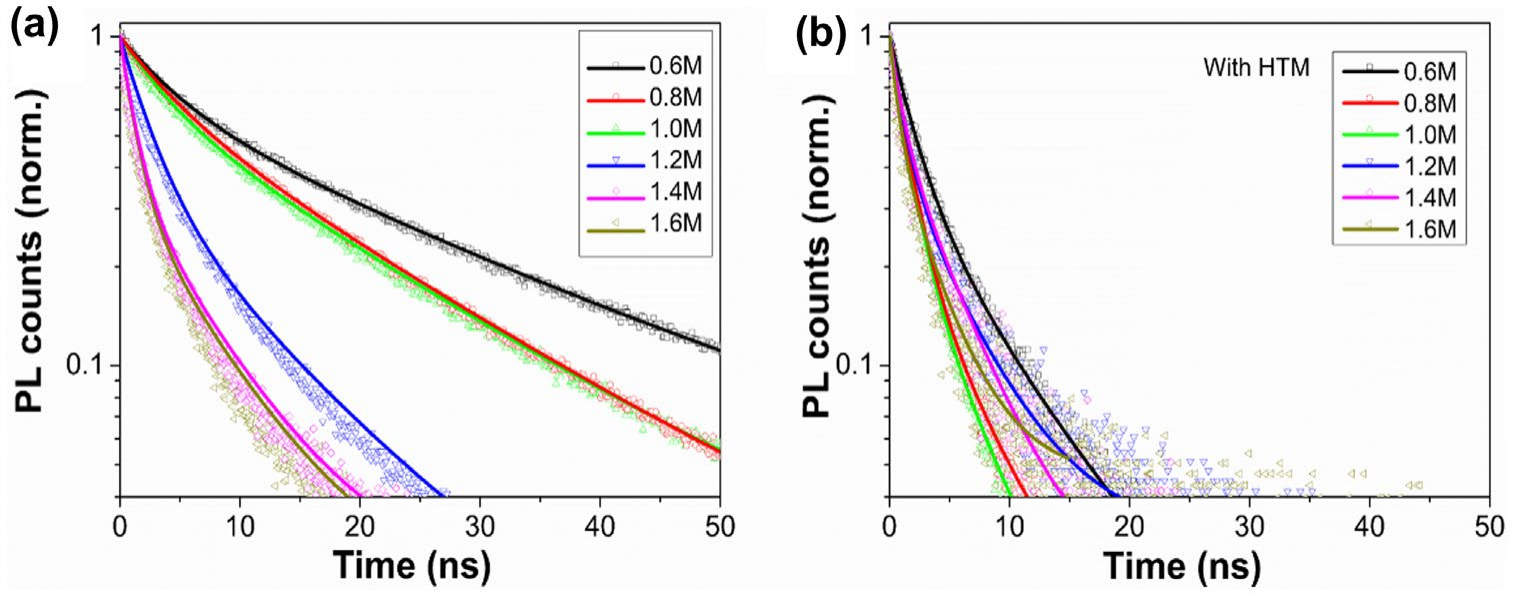
Time-resolved PL spectra of (a) FTO/bl-TiO_2_/mp-TiO_2_ /CH_3_NH_3_PbI_3_ films and (b) FTO/bl-TiO_2_/mp-TiO_2_/CH_3_NH_3_PbI_3_/HTM films as a function of *C*_(PbI2)_.

To further analyze the steady-state PL measurements and investigate the quenching efficiency of these *C*_(PbI2)_-dependent morphologies, time-resolved PL spectra (Figure 4(a)) were characterized. It has been demonstrated that there are two general mechanisms for recombination in bare CH_3_NH_3_PbI_3_ thin films, which can be described by a rate equation:

(2) $$\text{d}n/\text{d}t=An-{Bn}^{2}$$

where *n* is the excited carrier density, *A* and *B* represent the single-carrier trapping rate and the two-carrier radiative recombination coefficient, respectively [42,43]. In particular, the origin of the PL is driven by a radiative two-carrier (non-geminate) recombination process involving electrons and holes [38,44]. In bare semiconductor film, the carrier trapping dynamics is dominated by non-radiative recombination at energetically favored sites such as impurity sites and discrete defect at the bulk and interfaces of the material. Meanwhile, in the FTO/bl-TiO_2_/mp-TiO_2_/CH_3_NH_3_PbI_3_ film, the interface between TiO_2_ and CH_3_NH_3_PbI_3_ capping layer would lead to a strong decrease of the PL intensity by band bending at the interface, which can form a depletion region where charges are effectively quenched [45,46].

In our work, the PL lifetime was fitted with a bi-exponential function including a fast decay at early times followed by a slow decay process. When the photocarrier density (n) is large enough, in which condition discrete defect and impurity sites are saturated and the radiative rate is fast, the effective PL lifetime derived from Equation (3) can be approximately written as:[45]

(3) 1/τ=A+Bn

**Figure 5.**
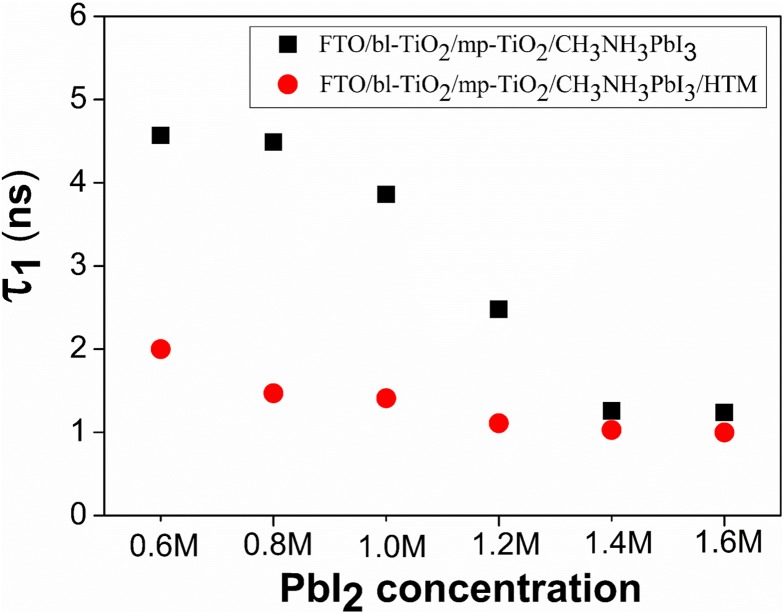
The radiative decay lifetime (τ_1_) determined by fitting the time-resolved PL spectra of FTO/bl-TiO_2_/mp-TiO_2_/CH_3_NH_3_PbI_3_ film with or without an HTM layer as a function of *C*_(PbI2)_.

This expression reproduces well the related parameters of PL decay shown in Table S1. It should be noticed that the laser beam of 405 nm with maximum fluence used here was strong enough to saturate the intermediate states and fully penetrate the perovskite films. We believe that the fast decay process is the result of radiative decay, which is strongly n-dependent as illustrated in Equation (3), reflecting the quenching efficiency of band bending at the interface. And the slow decay process is the result of carrier trapping mechanism, reflecting the non-radiative recombination via intermediate states [40]. For the samples of 0.6 M to 1.6 M, the radiative decay lifetime (τ_1_) gradually decreased from 4.57 to 1.24 ns (Figure 5), which indicated a more effective charge dissociation at higher *C*_(PbI2)_. As evidenced in Figures 1 and 3(b), the increase of *C*_(PbI2)_ centralized grain size distributions and improved the crystallinity of the perovskite thin films, facilitating the free carrier diffusion and enhancing the charge quenching efficiency.

To identify whether the charge quenching efficiency is complete at the TiO_2_/CH_3_NH_3_PbI_3_ interface, and to probe the charge recombination behavior in real devices, a spiro-OMeTAD HTM film containing LiTFSI and TBP dopants was fabricated onto the CH_3_NH_3_PbI_3_ capping layer and the corresponding PL decay was characterized (Figure 4(b)). The existence of an HTM layer atop the perovskite significantly facilitated the fast radiative recombination rate from 4.57 ns to 2.00 ns of sample of 0.6 M (Figure 5 and Table S1), indicating strong quenching efficiency at the interface between CH_3_NH_3_PbI_3_ and HTM layer, which was also demonstrated by Xing et al. [47] and Stranks et al. [33]. This suggests that the photo-generated free charges have not been fully quenched by the TiO_2_/CH_3_NH_3_PbI_3_ interface at *C*_(PbI2)_ of 0.6 M. For sample of 1.4 M, the fast decay lifetime decreased from 1.26 ns to 1.03 ns after adding an HTM layer. This little variation in the fast decay lifetime indicated that the charge quenching was mostly completed at the TiO_2_/CH_3_NH_3_PbI_3_ interface. It could explain the high efficiency of hole conductor-free CH_3_NH_3_PbI_3_/TiO_2_ heterojunction solar cells [48,49]. The slow decay process (τ_2_) representing carrier trapping mechanism was not analyzed here since the non-radiative recombination via intermediate states was strongly influenced by the crystallinity, discrete defect, impurity sites and film thickness of the CH_3_NH_3_PbI_3_ thin films.

**Figure 6.**
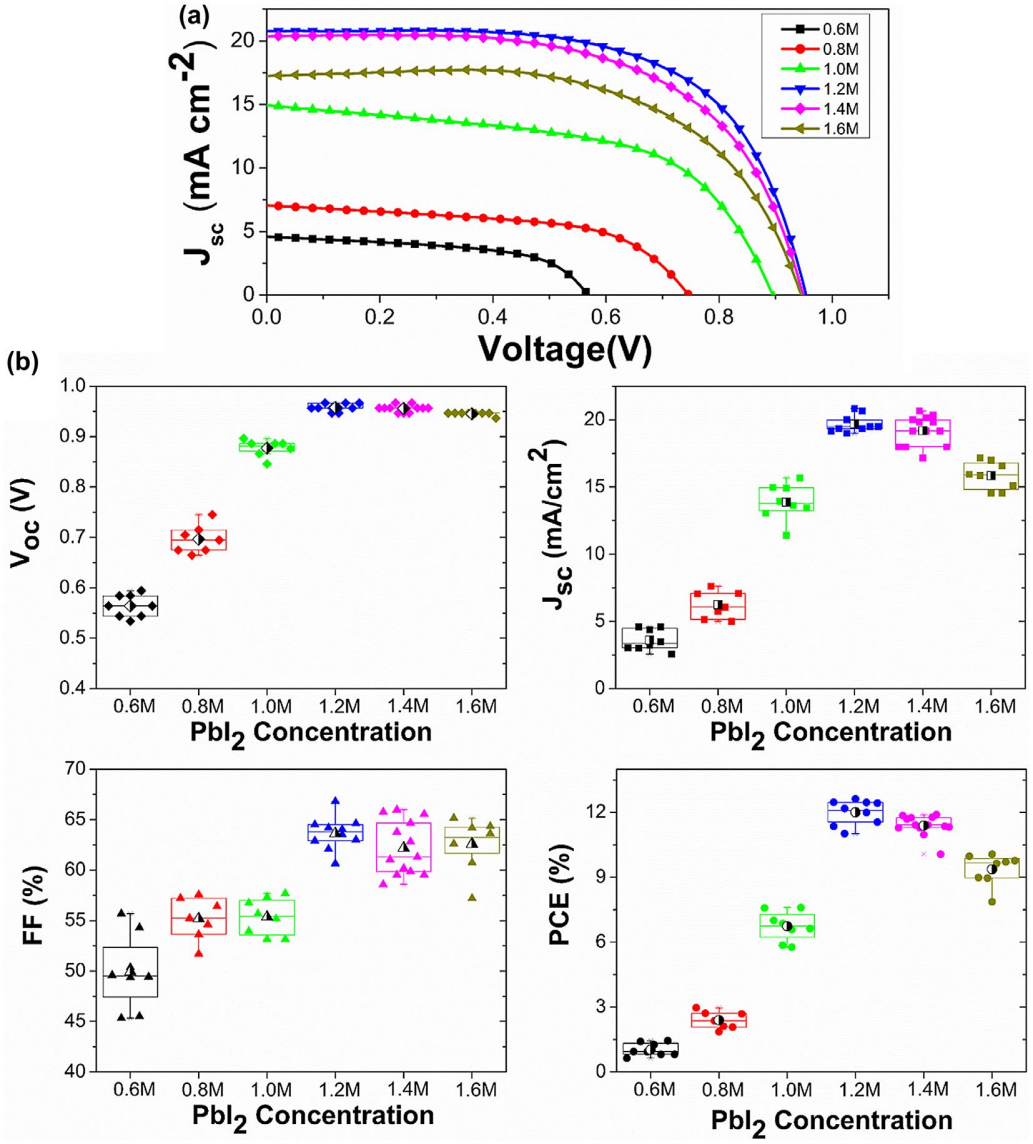
(a) *J*–*V* curves (under AM 1.5 G illumination of 1000 W m^–2^ intensity, active area of 6 mm^2^) as a function of *C*_(PbI2)_. (b) A comparison of photovoltaic parameters of devices exhibiting *C*_(PbI2)_-dependent morphologies. The half-black dots inside the box show the average values of all the samples.

The photovoltaic performance for perovskite solar cells with typical *C*_(PbI2)_-dependent morphologies are shown in Figure 6 and the detailed *J*−*V* characteristics of studied solar cells are tabulated in Table S2. To assure reliability in the measurements, at least eight devices at each condition were investigated. When 0.6 M PbI_2_ was used, the PCE was rather poor (~1%). By increasing the concentration of PbI_2_, the efficiency was first improved to 12.0% at *C*_(PbI2)_ of 1.2 M, and then decreased to 9.4%. The best performance of 12.6% was obtained at *C*_(PbI2)_ of 1.2 M with a short-circuit photocurrent density (*J*_sc_) of 20.8 mA cm^–2^, an open-circuit Voltage (*V*_oc_) of 947 mV, and a fill factor (*FF*) of 0.64, respectively. The *J*_sc_, *V*_oc_ and *FF* performed the same trend as the PCE. In particular, *J*_sc_ first improved from 3.6 to 19.7 mA cm^–2^ when the *C*_(PbI2)_ increased from 0.6 to 1.2 M, which is consistent with the enhanced absorption shown in Figure S5. Meanwhile, at low *C*_(PbI2)_ the insufficient charge carrier extraction across this heterogeneous interface as illustrated in PL decay measurement would cause a poor charge collection efficiency and further lead to a drop of *J*_sc_. However, further increasing *C*_(PbI2)_ from 1.2 to 1.6 M reduced *J*_sc_ from 19.7 to 15.8 mA cm^–2^ despite the enhanced absorption. This was attributed to the reduced perovskite coverage and the rough surface at high *C*_(PbI2)_ as shown in Figure 1, which indicated a less effective charge collection. IPCE spectra of the devices were tested from 300 nm to 830 nm as shown in Figure S6, and the integrated current density derived from the IPCE spectra corresponded well to the measured value from *J*−*V* measurement under simulated sunlight. It is worth noting that the great enhancement of IPCE spectra from 1.0 M to 1.2 M was supposed to be caused by the increased charge transporting efficiency across the device. The optical absorbance of 1.2 M perovskite film was very close to that of 1.2 M as shown in Figure S5(c), which indicated that the fairly equal amount of photogenerated charges were produced in the 1.0 and 1.2 M device. Furthermore, the increased charge transporting efficiency benefited from the smooth perovskite film produced by the disappearance of abnormal big perovskite crystals and also the increased thickness of perovskite layer [50].

*V*_oc_ increased steadily from 564 to 957 mV when *C*_(PbI2)_ increased from 0.6 to 1.2 M. The low *V*_oc_ indicates a charge recombination across the interface between ETM, CH_3_NH_3_PbI_3_ and HTM [51]. According to the *J*−*V* data shown in Figure 6 and Table S2, the *V*_oc_ decreased gradually from 957 to 945 mV when increasing *C*_(PbI2)_ from 1.2 M to 1.6 M. The *V*_oc_ decline trend is furthermore confirmed by the large amount of devices been made. The *V*_oc_ decrease revealed more serious charge recombination at higher *C*_(PbI2)_ above 1.2 M, which also contributed to decrease *J*_sc._ As evidenced in Figure 1, the abnormal grain growth of perovskite crystal (~400 nm) especially at low *C*_(PbI2)_ is attributed to an ultra-rough perovskite surface, which would be difficult for the HTM layer to fully cover the capping layer, leading to direct contact of CH_3_NH_3_PbI_3_ and Au. The *J*−*V* curves under different scan directions are shown in Figure S7. The hysteresis phenomena appeared in our measurement, which was common in not only planar structural PSCs but also mesoporous structural devices. Some groups demonstrated that the hysteresis phenomenon was strongly affected by perovskite crystal size, mesoporous TiO_2_ morphology and the thickness of perovskite capping layer [52–54], which had been speculated to originate from changes in absorber or contact conductivity, trapping/de-trapping of charge carriers, instinct ferroelectricity or ion migration [55,56]. Despite the hysteresis, the PCE of cells at different *C*_(PbI2)_ showed similar trends under forward scanning directions when comparing with the backward scanning data.

With comprehensive characterization combining FESEM, UV-vis, XRD, steady and time-resolved PL and *J*–*V* scans, we can see that a pinhole-free CH_3_NH_3_PbI_3_ capping layer with centralized grain sizes is critical for sufficient light absorption, achieving good interface properties by adequately extracting photo-generated free charges and obtaining an excellent photovoltaic performance. Extraordinarily big CH_3_NH_3_PbI_3_ crystals are harmful for the holistic crystallinity and the film smoothness, and will lead to a decrease of charge collecting efficiency by the recombination of transporting electrons and holes.

## 4. Conclusions

The abnormal grain growth of CH_3_NH_3_PbI_3_ crystals by a TSD method was observed at a low concentration of PbI_2_ precursor. We propose that a grain ripening process takes place during the transformation from PbI_2_ to CH_3_NH_3_PbI_3_: the relatively high surface energy of small crystalline grains results in that grains grow or ‘ripen’ at the expense of other ones accompanied with non-uniform elements distribution. Photo-generated charge recombination dynamics in CH_3_NH_3_PbI_3_ film was clearly illustrated by a recombination model, showing that the charge dissociation efficiency quenched by the interface between CH_3_NH_3_PbI_3_ and TiO_2_ was enhanced when increasing *C*_(PbI2)_. The PCE of the devices was first improved from 0.6 to 1.2 M and then decreased, consisting of the variation of perovskite grain growth and morphology. The pinhole-free CH_3_NH_3_PbI_3_ capping layer with centralized grain sizes is crucial for harvesting sufficient light and achieving good interface properties and photovoltaic performance.

## Disclosure statement

No potential conflict of interest was reported by the authors.

## Supplemental data

Supplemental material is linked to the article DOI online at http://dx.doi//10.1080/14686996.2017.1298974.

## Funding

The authors acknowledge the financial support provided by the Projects of International Cooperation and Exchanges NSFC [51561145007] and the Ministry of Science & Technology, P.R.China: Sino-Italy International Cooperation on Innovation [2016YFE0104000].s

## Supplementary Material

Supplementary_Materials_Hong_Lin.pdf
