# Supplementary material for: CH3NH3PbI3 grain growth and interfacial properties in meso-structured perovskite solar cells fabricated by two-step deposition
Source: Sci Technol Adv Mater. 2017 Apr 10;18(1):253–62. doi: 10.1080/14686996.2017.1298974 (PMC5402745; doi:10.1080/14686996.2017.1298974)
Supplement: Supplementary_Materials_Hong_Lin.pdf [file tsta_a_1298974_sm1841.pdf]

Supplementary Materials

$CH_3NH_3PbI_3$ grain growth and interfacial properties in meso-structured perovskite solar cells fabricated by two-step deposition

Zhibo Yao[1], Wenli Wang[2], Heping Shen[1], Ye Zhang[1], Qiang Luo[1], Xuewen Yin[1], Xuezeng Dai[1], Jianbao Li[3,1], and Hong Lin[1,]*

[1] State Key Laboratory of New Ceramics & Fine Processing, School of Materials Science and Engineering, Tsinghua University, Beijing 100084, China

[2] National Engineering Laboratory for Modern Silk, College of Textile and Clothing Engineering, Soochow University, Suzhou 215123, China

[3] State Key Laboratory of Marine Resource Utilization in South China Sea, Materials and Chemical Engineering Institute, Hainan University, Haikou 570228, China

Corresponding Author

*E-mail: hong-lin@mail.tsinghua.edu.cn

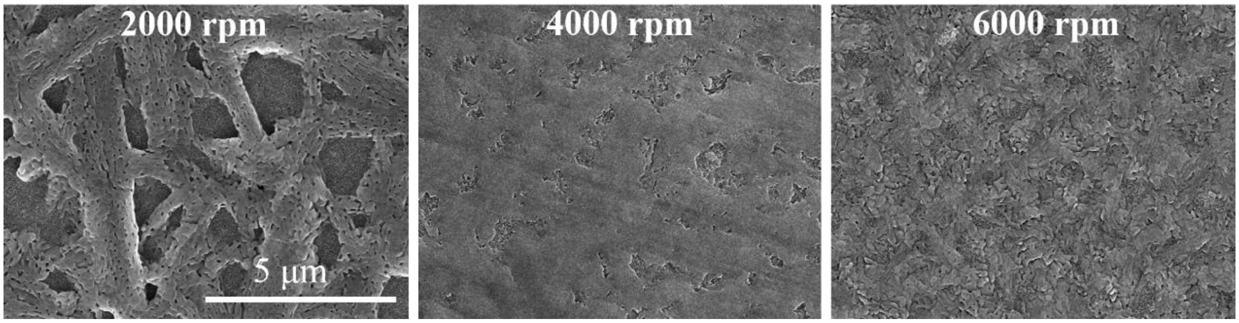

Figure S1. FESEM top views of FTO/ bl-TiO_2/ mp-TiO_2 / PbI_2 films fabricated with spinning speeds of 2000/ 4000/ 6000 rpm at $C_{(PbI2)}$ of 0.8 M.

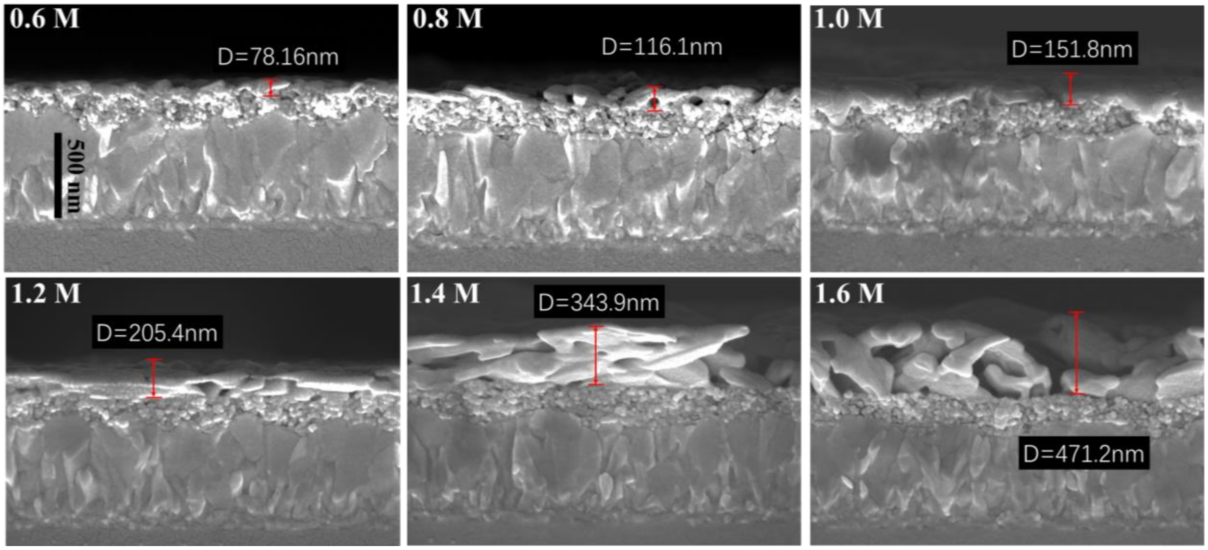

Figure S2. Cross-sectional SEM images of FTO/ bl-TiO_2/ mp-TiO_2 / PbI_2 films fabricated with $C_{(PbI2)}$ of 0.6 M to 1.6 M. The scale bar is 500 nm.

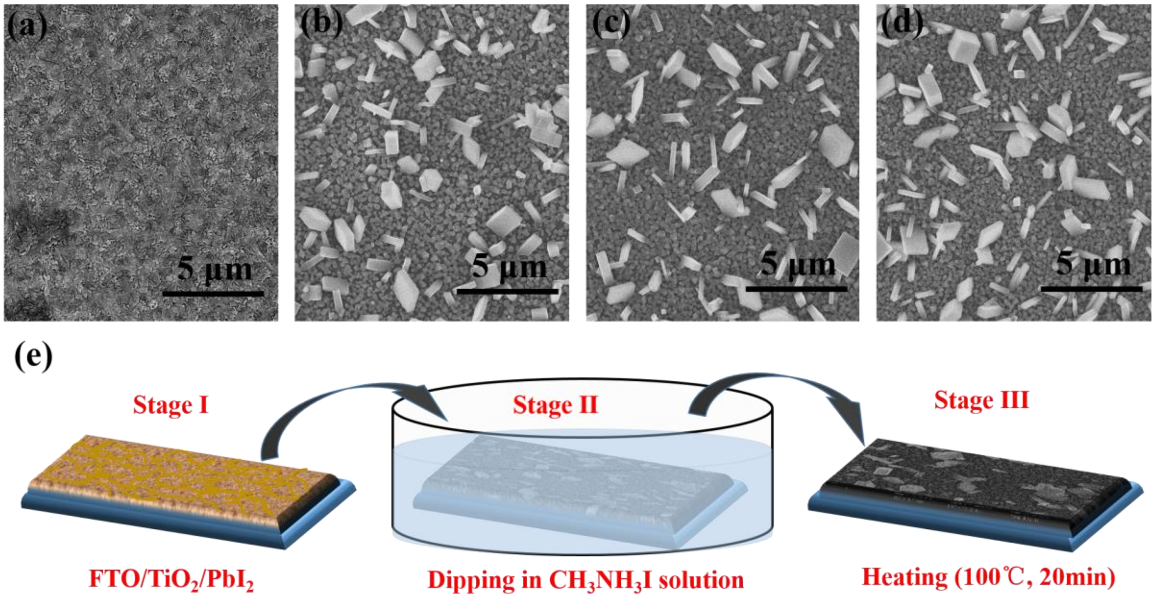

Figure S3. FESEM top views of (a) PbI_2 film, (b) $CH_3NH_3PbI_3$ films without annealing, and (c, d) $CH_3NH_3PbI_3$ films annealing at 100℃ for 10 min and 30 min respectively. The substrate is FTO/bl-TiO_2/mp-TiO_2 and the applied $C_{(PbI2)}$ is 0.8 M. (e) Schematic illustration of the growth process of the abnormal $CH_3NH_3PbI_3$ grains.

Energy dispersive spectroscopy (EDS) measurement:

We characterized the elements composition of the normal $CH_3NH_3PbI_3$ crystals and the abnormal ones with different morphologies using EDS on the SEM. We focused on the atomic ratio of different samples when concentrating the X-Ray on the specific spots (for example, the nanocubes in abnormal crystals).

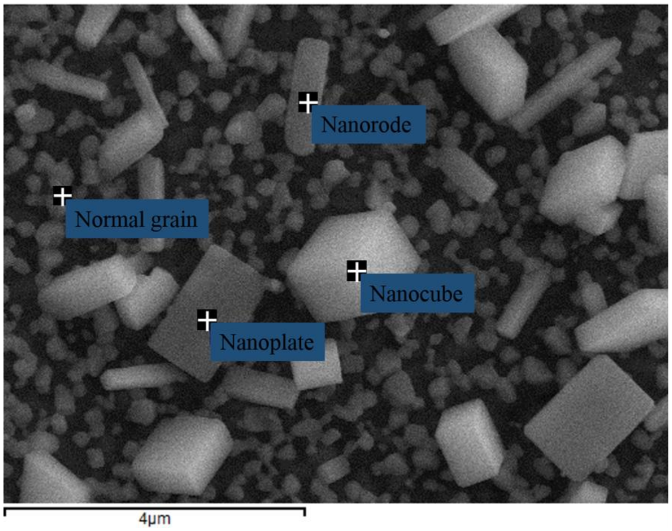

$CH_3NH_3PbI_3$ crystals	Pb: I atomic ratio
Nanocube	1: 2.80 ± 0.20
Nanoplate	1: 2.82 ± 0.14
Nanorode	1: 2.77 ± 0.12
Normal grain	1: 3.39 ± 0.31

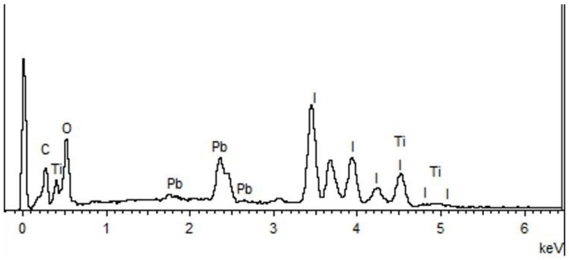

Figure S4. The Pb:I atomic ratio of different-shape perovskite crystals was calculated by the quantity analysis based on the EDS spectra. To increase the accuracy of the measurement, we scanned at least 4 spectra for the spots with the same morphology.

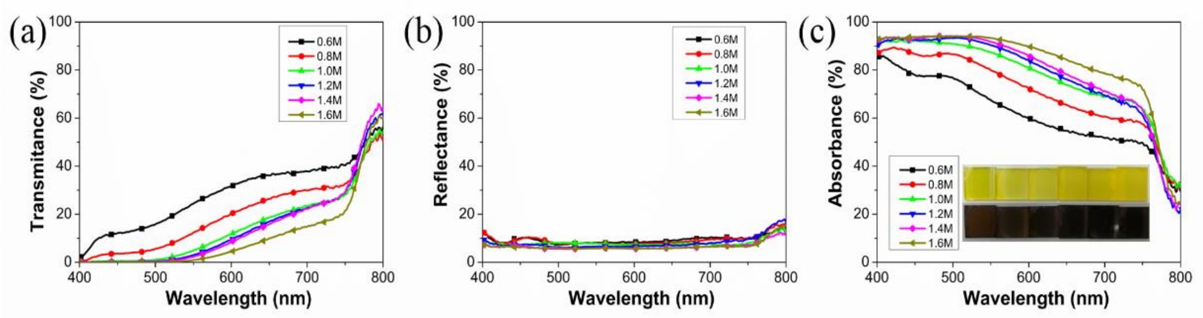

Figure S5. (a) UV-Vis transmittance, (b) reflectance and (c) absorbance spectra of FTO/bl-TiO_2/mp-TiO_2 /$CH_3NH_3PbI_3$ films as a function of $C_{(PbI2)}$. Inset of (c) depicts images of the spin-coated PbI_2 films (up) and the corresponding $CH_3NH_3PbI_3$ films (down) from left to right representing the $C_{(PbI2)}$ of 0.6 M to 1.6 M.

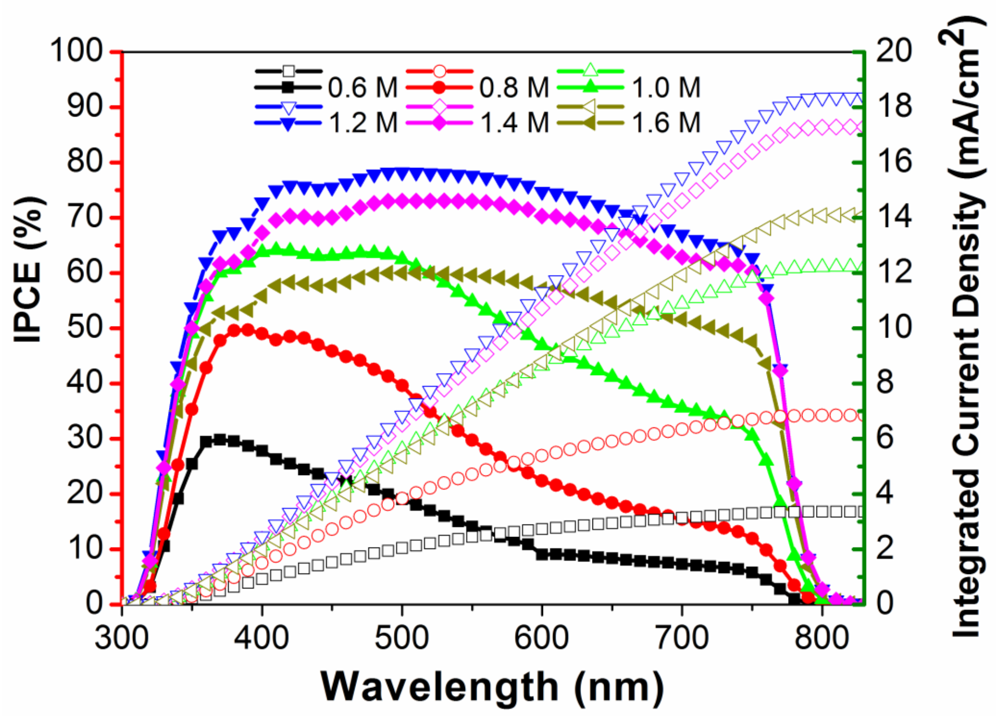

Figure S6. IPCE spectra and the relative integrated current density of the devices with the $C_{(PbI2)}$ of 0.6 M to 1.6 M.

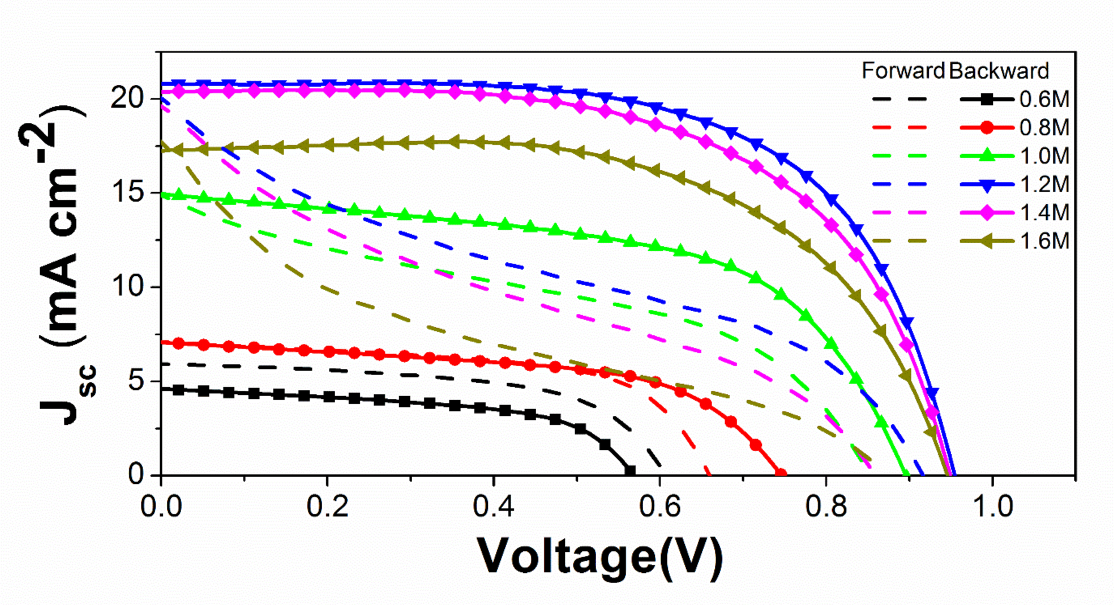

Figure S7. *J-V* curves with forward and backward scanning direction as a function of $C_{(PbI2)}$.

Table S1. Time-resolved photoluminescence characterization of the $C_{(PbI2)}$-dependent perovskite films.

HTM	Lifetime (ns)	0.6 M	0.8 M	1.0 M	1.2 M	1.4 M	1.6 M
None	τ_1	4.57	4.49	3.86	2.48	1.26	1.24
	τ_2	25.16	18.54	18.22	10.78	6.77	6.85
With	τ_1	2.00	1.47	1.41	1.11	1.03	1.00
	τ_2	7.66	5.56	5.00	5.00	4.56	3.46

Table S2. Photovoltaic performance of the studied solar cells with different $C_{(PbI2)}$-dependent perovskite morphologies collected by the average values of all the samples with standard derivation.

$C_{(PbI2)}$ (M)	J_{sc}(mA/cm2)	V_{oc}(mV)	*FF*(%)	PCE (%)
0.6	3.6±0.8	564±22	50±4	1.0±0.3
0.8	6.2±1.0	696±28	55±2	2.4±0.4
1.0	13.9±1.3	877±15	55±2	6.7±0.7
1.2	19.7±0.6	957±7	64±2	12.0±0.5
1.4	19.2±1.1	955±6	62±3	11.4±0.5
1.6	15.8±1.0	945±4	63±3	9.4±0.7
